# When Effort Meets Belief: A Moderated Mediation Model of Conscientiousness, Self-Efficacy, and Academic Engagement

**DOI:** 10.3390/bs16060850

**Published:** 2026-05-26

**Authors:** Kyueun Han, Min Young Kim

**Affiliations:** 1 College of Kyedang General Education, Sangmyung University, Seoul 03016, Republic of Korea; kyueun.han@smu.ac.kr; 2 Department of Psychology, Keimyung University, Daegu 42601, Republic of Korea

**Keywords:** conscientiousness, self-efficacy, academic engagement, internal locus of control, moderated mediation

## Abstract

Although conscientiousness is consistently associated with academic achievement, the psychological mechanisms underlying this relationship remain underexplored. We examine whether self-efficacy mediates the relationship between conscientiousness and academic engagement, and whether internal locus of control moderates this pathway. Our cross-sectional study included 1059 undergraduate students from a South Korean university, who completed validated measures of conscientiousness, self-efficacy, internal locus of control, and academic engagement. Using Hayes’ PROCESS macro, we conducted mediation and moderated mediation analyses. The findings support an indirect association between conscientiousness and academic engagement through self-efficacy. Bootstrapping analysis indicated a significant indirect effect, whereas the direct effect of conscientiousness on engagement became non-significant when self-efficacy was included in the model. Internal locus of control significantly moderated the self-efficacy–engagement pathway, and the indirect pathway was strongest among students with high internal locus of control beliefs. Conscientiousness influenced academic engagement indirectly through students’ confidence in their capabilities. This association was strongest among students who believed their academic outcomes were determined by their own efforts. Interventions should strengthen self-efficacy and internal control beliefs to promote sustained academic engagement among conscientious students.

## 1. Introduction

Academic engagement presents a central paradox in educational psychology: Why do students with equally high levels of conscientiousness display markedly different patterns of sustained engagement (Rieger et al., 2022; Trautwein et al., 2015)? Conscientiousness, defined as a personality trait encompassing organization, persistence, impulse control, and goal-directed behavior (Costa & McCrae, 1992), has consistently been identified as the most robust non-cognitive predictor of academic achievement across diverse populations and educational contexts (Mammadov, 2022; Meyer et al., 2024). Despite this strong association, a substantial portion of the variance in engagement among conscientious students remains unexplained (Richardson et al., 2012), indicating the need for a more nuanced theoretical examination.

Traditional explanations attribute the academic benefits of conscientiousness to heightened self-control and disciplined study habits (Rosidah et al., 2025). However, recent evidence challenges this view, suggesting that conscientiousness may influence engagement through mechanisms beyond effortful self-control.

We propose that academic self-efficacy serves as a key psychological pathway linking personal traits with academic engagement (Alethar et al., 2025). Conscientious students may succeed not only by working harder but also by cultivating strong self-confidence in their abilities. Self-efficacy predicts persistence after setbacks, adaptive help-seeking, and the use of deep-learning strategies, as well as outcomes for which effort alone may be insufficient (Usher et al., 2023). If conscientiousness operates primarily by fostering self-efficacy, it reframes the personality–achievement link from a behavioral to a motivational-cognitive mechanism. This view is supported by prior findings that conscientiousness predicts self-efficacy, which in turn enhances academic performance (Komarraju et al., 2009).

However, confidence alone may not translate into engagement. Locus of control, defined as the extent to which individuals believe that academic outcomes are determined by their own actions (internal) versus external forces (external), may shape this relationship. Students with a strong internal locus of control demonstrate a stronger link between self-efficacy and performance than those with an external orientation (Sagone & De Caroli, 2014; Suraj et al., 2024). This suggests that control beliefs act as a boundary condition, enabling self-efficacy to manifest as tangible engagement behavior.

While the bivariate relationships among conscientiousness, self-efficacy, and academic engagement have been examined, few studies have integrated personality, domain-specific beliefs, and attributional orientations within a single, comprehensive framework. This study addresses this gap by simultaneously testing self-efficacy as a mediator and internal locus of control as a boundary condition, offering a more thorough understanding of how stable traits and malleable beliefs jointly influence academic engagement.

Consequently, our study uses an integrated moderated mediation model to investigate the relationships among conscientiousness, self-efficacy, and academic engagement, with a particular focus on the role of internal locus of control. We address the following three questions:

(1) Does self-efficacy mediate the conscientiousness–engagement relationship?
(2) Does internal locus of control moderate the self-efficacy–engagement pathway?
(3) What are the theoretical and practical implications of supporting students at risk of disengagement despite high conscientiousness?

### 1.1. Theoretical Background

Within the five-factor model (Costa & McCrae, 1992), conscientiousness encompasses organization, persistence, impulse control, and goal-directed behavior, with facets including achievement-striving, self-discipline, and deliberation (Roberts et al., 2006). In academic contexts, this trait manifests as systematic study routines, timely completion of assignments, and sustained efforts toward long-term educational goals.

Empirical evidence for the predictive power of conscientiousness in academic achievement is both extensive and consistent. Meta-analysis findings indicate moderate positive associations between conscientiousness and academic performance, with corrected effect sizes typically ranging from 0.22 to 0.25 (Poropat, 2009). In a more recent synthesis of 267 independent samples (N = 413,000), Mammadov (2022) reported that conscientiousness remained a significant predictor of academic outcomes even after controlling for cognitive ability, accounting for approximately 28% of the explained variance. These results hold across educational levels, subject domains, and cultural contexts, reflecting the cross-cultural replicability of the Big Five model and robustness of conscientiousness as a non-cognitive predictor of performance.

Traditional theoretical accounts primarily attribute the academic benefits of conscientiousness to its behavioral manifestations. Dumfart and Neubauer (2016) identified conscientiousness as the strongest non-cognitive predictor of school achievement among adolescents, even after adjusting for intelligence. Similarly, meta-analytic reviews (e.g., Credé et al., 2010) have linked class attendance to college performance, although the association between conscientiousness and attendance is modest. These findings support the view that conscientiousness promotes success through consistent and disciplined behaviors.

However, recent theoretical developments have challenged this purely behavioral interpretation. Inzlicht and Roberts (2024) suggested that conscientious individuals may rely less on momentary self-control and more on proactive environmental structuring and habit formation, shifting the explanatory focus from effortful regulation toward motivational and cognitive processes.

These insights underscore the limitations of direct-effect models: some highly conscientious students may meet formal academic requirements without demonstrating deep intellectual engagement, suggesting that additional psychological resources are necessary to translate conscientiousness into meaningful engagement. A deeper understanding of these mediating mechanisms is essential for advancing theory and developing targeted interventions (Rieger et al., 2022).

#### 1.1.1. Self-Efficacy

The self-efficacy theory, articulated by Bandura (1977, 1997), offers a comprehensive framework for understanding how beliefs about one’s capabilities influence behavior, motivation, and achievement. Self-efficacy is defined as an individual’s assessment of their capability to execute the actions required for specific performance outcomes (Bandura, 1997); in educational settings, this manifests as students’ confidence in their ability to successfully attain desired learning goals (Bong & Skaalvik, 2003). For example, a student may demonstrate high self-efficacy in mathematics but low self-efficacy in writing, reflecting the contextual nature of their efficacy beliefs. Such specificity enhances the predictive validity for behavior in particular academic domains.

Bandura’s (1977) model identifies four primary sources of efficacy beliefs: enactive mastery experiences (the most influential), vicarious experiences, social persuasion, and physiological and affective states (Usher & Pajares, 2008). Of these, mastery experiences are most relevant to the present study, as conscientious students’ organized behaviors create opportunities for direct success that strengthen efficacy beliefs.

Self-efficacy shapes academic engagement through multiple pathways: students with stronger efficacy beliefs pursue more demanding coursework, persist longer in the face of difficulty, employ deeper cognitive strategies, and recover more readily from setbacks (Bandura, 1997; Schunk & Pajares, 2009; Zimmerman, 2000). From a theoretical standpoint, self-efficacy is well-positioned to mediate the conscientiousness–engagement relationship: conscientious students’ organized, goal-directed behaviors generate mastery experiences that strengthen efficacy beliefs, which in turn sustain engagement (Caprara et al., 2011).

Empirical evidence supports this mediating pathway. Caprara et al. (2011) found that academic self-efficacy fully mediated the relationship between conscientiousness and achievement, rendering the direct effect of conscientiousness non-significant. Stajkovic et al.’s (2018) meta-analysis confirmed that self-efficacy was a robust mediator that linked individual differences to performance-related outcomes. Collectively, these findings suggest that while conscientiousness provides a behavioral foundation for academic success, self-efficacy is the key psychological mechanism that translates this disposition into sustained engagement. We hypothesize the following:

**Hypothesis 1.** 
*Self-efficacy mediates the relationship between conscientiousness and academic engagement, such that conscientiousness enhances self-efficacy beliefs, which, in turn, lead to increased academic engagement.*

#### 1.1.2. Locus of Control

Locus of control, introduced by Rotter (1966) within social learning theory, describes individual differences in perceived control over life outcomes. Individuals with an internal locus of control believe that outcomes are primarily determined by their own actions, whereas those with an external locus of control attribute outcomes to factors such as luck, fate, or the influence of powerful others. Although the construct was initially considered unidimensional, recent research has supported a multidimensional view that varies across life domains, including academic contexts (Santokhie & Lipps, 2020).

Although locus of control operates across life domains, generalized internal control forms a dispositional foundation that shapes students’ approaches to learning and interpretation of academic outcomes. Students with a largely internal orientation are more likely to view outcomes, including academic ones, as contingent upon their efforts and strategies, believing sustained effort leads to desired outcomes and success is within their control. This belief fosters agency, responsibility, proactive learning behaviors, and greater persistence (Findley & Cooper, 1983). In contrast, students with an external orientation attribute academic outcomes to uncontrollable factors such as teacher bias or luck, which can lead to diminished motivation and, in extreme cases, learned helplessness (Weiner, 1985).

A substantial body of evidence supports the positive association between an internal locus of control and academic achievement. Meta-analysis findings indicate that internal orientation predicts higher achievement across various educational levels and contexts (Findley & Cooper, 1983; Ng et al., 2006). Recent studies suggest that this orientation amplifies the relationship between self-efficacy and academic performance (Suraj et al., 2024). This effect appears to be mediated by self-regulatory behaviors; internally oriented students are more likely to engage in time management, strategic planning, and proactive help-seeking, perceiving these actions as effective means to influence outcomes (Gifford et al., 2006).

The theoretical rationale for locus of control as a moderator is its conceptual complementarity with self-efficacy. While self-efficacy reflects beliefs about one’s capability to perform a task, locus of control reflects beliefs about whether such performance translates into desired outcomes. Students may possess high domain-specific self-efficacy with a primarily external locus of control, believing that life outcomes are largely determined by factors beyond their control. This generalized attributional stance shapes the interpretation of academic outcomes, dampening the motivational utility of self-efficacy. If effort is perceived as irrelevant to outcomes, the incentive to sustain engagement diminishes.

As noted above, optimal engagement requires both strong efficacy beliefs and internal control orientations, with the latter serving as a generalized outcome expectation that strengthens the motivational impact of self-efficacy (Bandura, 1997).

Mechanistically, internal locus of control may moderate the self-efficacy–engagement link by (1) reinforcing the perceived connection between effort and outcomes, thereby motivating sustained investment; (2) reducing cognitive dissonance between high-capability beliefs and the perceived randomness of outcomes; and (3) promoting resilience following failure, as internally oriented students attribute setbacks to controllable factors, thereby protecting self-efficacy from erosion.

These empirical findings support the proposed moderating hypothesis. Hsieh et al. (2007) reported that college students with an internal locus of control demonstrated stronger associations between self-efficacy and achievement than those with an external orientation. Similarly, Schunk and Zimmerman (2007) proposed that efficacy-enhancing interventions were more effective when individuals believed that outcomes depended on their own actions, as such beliefs encouraged the adoption and sustained use of self-regulatory strategies. Thus, we hypothesize:

**Hypothesis 2.** 
*Internal locus of control moderates the relationship between self-efficacy and academic engagement, such that the positive association is stronger among students with a high internal locus of control.*

#### 1.1.3. Proposed Model

The proposed moderated mediation model integrates personality, socio-cognitive, and attribution theories to explain how conscientiousness fosters academic engagement. This framework addresses two key questions: Why does conscientiousness predict achievement without requiring constant willpower, and why do equally conscientious students differ in their engagement levels?

The model relies on the following three principles. First, personality traits indirectly influence behavior through proximal psychological mechanisms. Conscientiousness creates conditions conducive to the development of self-efficacy, whereas self-efficacy, a domain-specific belief system, directly drives engagement. Second, the effectiveness of psychological mechanisms depends on broader attributional frameworks; internal control strengthens the self-efficacy–engagement link, whereas external control weakens it. Third, academic engagement emerges from the dynamic interactions between stable traits (conscientiousness), malleable beliefs (self-efficacy), and attributional orientations (locus of control).

The proposed model posits complete mediation through self-efficacy, suggesting that conscientiousness does not directly cause engagement but fosters conditions for efficacy growth. This aligns with Inzlicht and Roberts’ (2024) view that conscientiousness functions through strategic environmental structuring, such as maintaining consistent schedules and organized materials, which generate mastery experiences that build self-efficacy and ultimately drive engagement.

The moderating role of internal locus of control becomes clearer when self-efficacy is the most influential factor. Under conditions of high internal control, conscientiousness builds self-efficacy, which translates strongly into engagement. Under low internal control, this pathway weakens, as students doubt that their efforts determine outcomes.

This integrated model addresses several theoretical issues. The conscientiousness–engagement paradox (why some conscientious students show low engagement) may be explained by insufficient self-efficacy or external control orientations. The efficacy–performance gap (why high self-efficacy sometimes fails to produce engagement) may reflect a lack of attributional support. Finally, the intervention-failure problem (why single-target interventions often underperform) suggests that interventions should simultaneously address conscientiousness, self-efficacy, and locus of control.

From a developmental standpoint, the relative contributions of these components may theoretically vary across educational stages, with the mediating role of self-efficacy and the moderating role of locus of control potentially becoming more salient as academic demands intensify. However, these propositions remain speculative within the present cross-sectional framework. Longitudinal research is needed to determine whether these components mutually reinforce one another over time and whether such self-sustaining patterns emerge in practice. We hypothesize the following:

**Hypothesis 3.** 
*The indirect effect of conscientiousness on academic engagement through self-efficacy is moderated by internal locus of control, such that the mediation pathway is strongest when the internal locus of control is high.*

**Figure 1:**
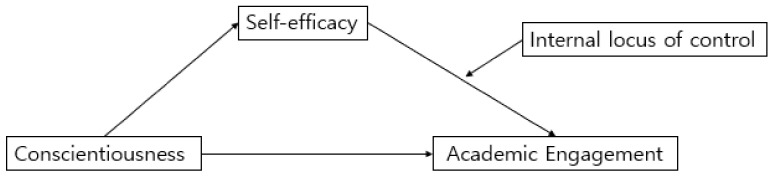
Proposed model showing that conscientiousness influences academic engagement through self-efficacy, moderated by internal locus of control.

In summary, this theoretical framework integrates personality, socio-cognitive, and attribution perspectives into a comprehensive moderated mediation model. By examining self-efficacy as a psychological pathway and internal locus of control as a potential boundary condition, the model offers a framework to reinterpret long-standing paradoxes in the literature and suggests directions for future research and interventions. The hypothesized relationships are depicted in Figure 1, which presents the tested model.

## 2. Materials and Methods

### 2.1. Participants

A total of 1059 undergraduate students participated in the study. All participants were enrolled in the course “Modern Society and Psychology” at Sangmyung University. Participation was voluntary, and no personal information was collected or used. This procedure ensured participant anonymity and compliance with the ethical standards of research. The study was submitted to the Institutional Review Board (IRB) of Sangmyung University (application number: IRB-SMU-S-2023-3-006) and, following formal review, was granted exemption (exemption approval number: SMIRB: ex-2023-004).

### 2.2. Procedure

Participants were recruited through convenience sampling from students enrolled in “Modern Society and Psychology.” No specific inclusion or exclusion criteria were applied, other than that participants were adults and could respond in the language in which the measurement instruments were administered. Participation was open to all students enrolled in the course. Recruitment was conducted via an online announcement posted on e-Campus, the university’s course-based learning management system. Students were informed that participation would serve as a practical learning experience in psychological research methods and that the study results would be shared as part of the course.

To minimize potential pressure arising from the instructor–student relationship, several safeguards were implemented. Participation was entirely voluntary, and students were not obligated to participate. Participation or non-participation had no impact on course grades or any other academic evaluation, and this was explicitly communicated to students at the time of recruitment. Responses were collected via an anonymous online survey, ensuring that the research team could not identify which students had participated. Students were also informed that only aggregate findings, not individual responses, would be shared in the course context. Despite these safeguards, we acknowledge that subtle social-desirability pressures or demand characteristics cannot be fully ruled out; this limitation is addressed in Section 4.

The online survey began with an introductory page that detailed the study’s purpose, the institution’s affiliation, and its ethical considerations. This page also emphasized the voluntary nature of participation, explained that the results could be disseminated through academic publication, and informed consent was obtained from each participant. The questionnaire was administered in Korean and required approximately 20 min to complete. Data collection procedures incorporated multiple safeguards to ensure data security and maintain confidentiality.

### 2.3. Measures

The questionnaire included validated scales that assessed four constructs: conscientiousness, academic engagement, self-efficacy, and internal locus of control. These were selected to test a moderated mediation model wherein the internal locus of control moderated the indirect effect of conscientiousness on academic engagement via self-efficacy. All items were presented in an online format, and responses were recorded through the survey platform.

### 2.4. Internal Locus of Control

Internal locus of control was assessed using the Internal Scale of the Multidimensional Locus of Control Scale (Levenson, 1973). This scale comprises eight items that measure the extent to which individuals believe that they have personal control over life events. Items were rated on a 7-point Likert scale (1 = *Strongly Disagree* to 7 = *Strongly Agree*), with higher scores indicating stronger internal control beliefs. Prior research has demonstrated the acceptable reliability and construct validity of this scale. Example items include “I can pretty much determine what will happen in my life” and “My own actions determine my life.”

In the present study, Cronbach’s α was 0.62, which falls below the conventional threshold for adequate internal consistency (α ≥ 0.70). This relatively low reliability likely reflects the scale’s brevity (8 items) and the inherent heterogeneity of the locus of control construct, which encompasses diverse life domains. The implications of this reliability level for interpreting our moderation results are addressed in Section 4. Notably, this scale assesses generalized internal control beliefs rather than academic-specific control beliefs. We selected the generalized measure on theoretical grounds, arguing that dispositional control orientation provides a broad attributional framework that shapes how individuals interpret outcomes across domains, including academic contexts (see Section 1.1.2). The implications of this measurement choice are also addressed in Section 4.

### 2.5. Academic Engagement

Academic engagement was measured using a six-item scale developed by Kwon (2008) to assess students’ enthusiasm and active involvement in learning and academic activities. Items were rated on a 5-point Likert scale (1 = *Strongly Disagree* to 5 = *Strongly Agree*), with higher scores indicating greater academic engagement. The scale captures both affective engagement (e.g., “I get excited when I learn something new”) and behavioral engagement (e.g., “When I want to know something, I immediately search for it through the Internet and library” and “Whenever there is a public lecture, I always attend and listen”). Several items reflect a relatively broad operationalization of engagement, extending to self-directed academic curiosity and enrichment behaviors beyond formal instructional contexts. Accordingly, the scale is best understood as capturing a broad conceptualization of academic engagement rather than engagement in the narrower motivational sense emphasized in some contemporary frameworks (Fredricks et al., 2004; Skinner et al., 2009). Cronbach’s α in this study was 0.83.

### 2.6. Conscientiousness

Conscientiousness was measured using a shortened version of the Big Five Inventory (John & Srivastava, 1999), which comprises five items from the original seven-item conscientiousness subscale. The items assessed tendencies toward organization, responsibility, and dependability. Items were rated on a 7-point Likert scale (1 = *Strongly Disagree* to 7 = *Strongly Agree*), with higher scores indicating greater conscientiousness. The Big Five Inventory has strong psychometric properties across diverse populations. In this study, Cronbach’s α was 0.71.

### 2.7. Self-Efficacy

Self-efficacy was measured using a modified version of the Self-Efficacy Scale, initially developed by Sherer et al. (1982) based on Bandura’s (1977) theory and later adapted for Korean populations by Kim and Park (2001). The scale contains 19 items across three subfactors: efficacy, self-confidence, and perseverance. Items were rated on a 5-point Likert scale (1 = *Strongly Disagree* to 5 = *Strongly Agree*), with higher scores indicating higher self-efficacy. This scale has been widely used in educational and psychological research, with strong evidence of validity. In this study, Cronbach’s α was 0.88.

### 2.8. Data Analysis

All statistical analyses were conducted using SPSS Windows software, version 28.0, with the PROCESS macro, version 4.0 (Hayes, 2017). Descriptive statistics and Pearson’s correlation coefficients were calculated to examine the relationships among the study variables. To provide a preliminary assessment of potential common method bias, we conducted Harman’s single-factor test by entering all measurement items into an exploratory factor analysis with a fixed single-factor solution. The single factor accounted for 25.47% of the total variance, which is below the 50% threshold often cited in the literature (Podsakoff et al., 2003). However, Harman’s single-factor test is widely recognized as a limited diagnostic. It has low sensitivity for detecting method bias, does not partial out method variance from substantive relationships, and cannot rule out shared-method inflation when all focal variables are measured from the same respondents using the same self-report format at a single time point (Podsakoff et al., 2003; Fuller et al., 2016). Therefore, we do not treat this test as evidence of severe common method bias, but as a demonstration that such bias is absent. We discuss the implications of this measurement context in Section 4. To test the hypothesized mediation model, we employed the PROCESS Model 4 to determine whether self-efficacy mediated the relationship between conscientiousness and academic engagement. For the moderated mediation analysis, we used the PROCESS Model 14 to test whether internal locus of control moderated the indirect effect of conscientiousness on academic engagement through self-efficacy. All 1059 participants provided complete responses on the focal variables, with no exclusions. Accordingly, all analyses, including the mediation (Section 3.2) and moderated mediation (Section 3.3) models, were conducted on the full sample (*N* = 1059), with no missing data or listwise deletion required. We used Bootstrapping procedures with 5000 resamples to generate bias-corrected confidence intervals for indirect and conditional effects. Statistical significance was set at *p* < 0.05 for all analyses. The moderated mediation effect was considered significant if the confidence interval for the index of moderated mediation did not include zero. Self-efficacy and internal locus of control were mean-centered prior to the moderated mediation analysis to facilitate the interpretation of interaction effects and to reduce non-essential multicollinearity between the interaction term and its component variables (Aiken & West, 1991). Variance inflation factors (VIFs) for all predictors ranged from 1.00 to 1.53, confirming that multicollinearity was not a concern.

## 3. Results

### 3.1. Descriptive Statistics and Correlations

Table 1 presents the descriptive statistics and intercorrelations for the primary variables: conscientiousness, internal locus of control, self-efficacy, and academic engagement. Conscientiousness and self-efficacy showed a moderate to strong positive correlation (*r* = 0.53, *p* < 0.01), indicating a substantial association between the two constructs. Conscientiousness was also moderately and positively correlated with internal locus of control (*r* = 0.36, *p* < 0.01), suggesting a meaningful association between personal control beliefs and conscientious tendencies.

Self-efficacy was significantly correlated with both internal locus of control (*r* = 0.44, *p* < 0.01) and academic engagement (*r* = 0.42, *p* < 0.01). These relationships indicate that students with higher self-efficacy are more likely to feel in control of their academic outcomes and actively engage in learning. The weakest significant correlation was observed between conscientiousness and academic engagement (*r* = 0.22, *p* < 0.01). This finding indicates conscientiousness alone was a relatively modest predictor of engagement, potentially requiring mediating factors, such as self-efficacy, to strengthen its impact.

### 3.2. Mediation Analysis of Self-Efficacy

The results are summarized in Table 2. In the initial model (Model 1), conscientiousness significantly predicted academic engagement (*B* = 0.18, *t* = 7.29, *p* < 0.001), accounting for 5% of the variance (*R*^2^ = 0.05, *F*(1, 1057) = 53.20, *p* < 0.001). In Model 2, conscientiousness significantly predicted self-efficacy (*B* = 0.32, *t* = 20.06, *p* < 0.001), explaining 28% of the variance (*R*^2^ = 0.28, *F*(1, 1057) = 402.33, *p* < 0.001). In the full mediation model (Model 3), self-efficacy significantly predicted academic engagement (*B* = 0.58, *t* = 12.91, *p* < 0.001). In contrast, the direct effect of conscientiousness on academic engagement was non-significant (*B* = −0.01, *t* = −0.10, *p* = 0.918). This model explained 18% of the variance in academic engagement (*R*^2^ = 0.18, *F*(2, 1056) = 114.10, *p* < 0.001).

Bootstrapping analysis with 5000 resamples revealed conscientiousness had a significant indirect effect on academic engagement through self-efficacy (*B* = 0.18, 95% CI [0.15, 0.22]) because the confidence interval did not include zero. These findings are consistent with an indirect association between conscientiousness and academic engagement operating through self-efficacy, supporting Hypothesis 1. Following current methodological guidelines (Hayes, 2022; Zhao et al., 2010), this conclusion rests on the significance of the bootstrapped indirect effect rather than on the non-significance of the direct effect. Given the cross-sectional design, this pattern should be interpreted as a statistical association consistent with a mediating role of self-efficacy rather than as evidence of a causal mediation process.

### 3.3. Moderated Mediation Model

Table 3 presents the results of the moderated mediation analysis examining whether internal locus of control moderates the indirect effect of conscientiousness on academic engagement through self-efficacy. Self-efficacy and internal locus of control were mean-centered prior to forming the interaction term to facilitate interpretation and reduce non-essential multicollinearity (Aiken & West, 1991). We conducted the analysis using Model 14 of Hayes’ PROCESS macro. VIFs for all predictors ranged from 1.00 to 1.53, well below the conventional threshold of 5 (O’Brien, 2007), indicating that multicollinearity was not a concern.

In the first stage, conscientiousness significantly predicted self-efficacy (*B* = 0.32, *t* = 20.06, *p* < 0.001), accounting for 28% of the variance (*R*^2^ = 0.28, *F*(1, 1057) = 402.33, *p* < 0.001).

In the second stage, self-efficacy significantly predicted academic engagement (*B* = 0.57, *t* = 12.04, *p* < 0.001), and the interaction between self-efficacy and internal locus of control was also significant (*B* = 0.13, *t* = 2.67, *p* < 0.01). This result implied internal locus of control moderated the relationship between self-efficacy and engagement. The main effect of internal locus of control on academic engagement was non-significant (*B* = 0.03, *t* = 0.72, *p* = 0.469). The full model explained 18% of the variance in academic engagement (*R*^2^ = 0.18, *F*(4, 1054) = 59.33, *p* < 0.001).

Bootstrapping with 5000 resamples supported this finding. The index of moderated mediation was 0.04 (*SE* = 0.02, 95% CI [0.01, 0.08]); the exclusion of zero from the confidence interval confirmed statistical significance.

**Figure 2:**
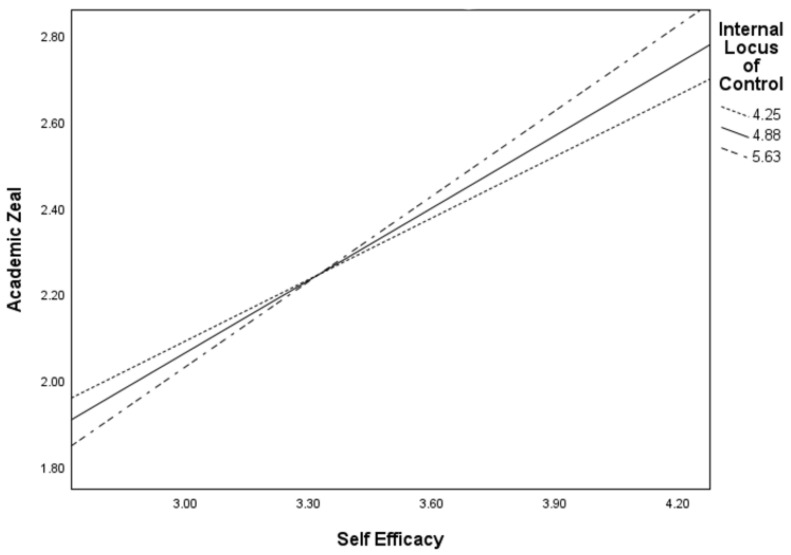
Moderating effect of internal locus of control on the relationship between self-efficacy and academic engagement.

Conditional indirect-effect analysis revealed that the indirect effect of conscientiousness on academic engagement through self-efficacy varied across levels of internal locus of control. Specifically, the indirect effect was significant at low (Internal = 4.25, Effect = 0.15, 95% CI [0.11, 0.19]), moderate (Internal = 4.88, Effect = 0.18, 95% CI [0.14, 0.21]), and high (Internal = 5.63, Effect = 0.21, 95% CI [0.16, 0.26]) levels of internal locus of control. The mediation pathway was strongest when internal locus of control was high, indicating the positive association between conscientiousness and academic engagement, operating through self-efficacy, was strongest among students who maintained strong internal control beliefs (see Figure 2).

## 4. Discussion

This study tested a moderated mediation model integrating personality, socio-cognitive, and attribution theories to explain how conscientiousness influenced academic engagement. This integrated model advances the literature, providing evidence of an association between conscientiousness and engagement consistent with an indirect pathway through self-efficacy, which varies with internal control beliefs. The simultaneous examination of these processes in a large undergraduate sample is a rare empirical synthesis of personality, socio-cognitive, and attribution perspectives, thus offering novel insights into the conditional mechanisms of academic motivation.

The results supported all three hypotheses. Specifically, the bootstrapped indirect effect of conscientiousness on engagement through self-efficacy was statistically significant, and this indirect effect was stronger among students with higher internal locus of control. This pattern is consistent with, though it does not establish, an indirect-effect interpretation that complements traditional direct-effect models of conscientiousness and academic outcomes. Conscientious students may cultivate conditions fostering academic success, which then support stronger self-efficacy beliefs and engagement. Given the cross-sectional design, these relationships should be interpreted as statistical associations rather than causal processes. This pattern of associations aligns with contemporary perspectives emphasizing that self-regulation and perceived control play a central role in translating broad personality traits, such as conscientiousness, into academic behaviors and outcomes (Caprara et al., 2011; Zimmerman, 2000), thereby extending earlier theoretical models (Bandura, 2006; Roberts et al., 2006).

The moderation results further identified internal locus of control as a critical boundary condition for the efficacy–engagement link. Students who held stronger generalized beliefs that outcomes across life domains depended on their own actions exhibited the strongest positive association between self-efficacy and engagement. This pattern is consistent with the view that generalized internal control functions as a broad attributional lens that shapes the interpretation of academic outcomes, although the magnitude of this effect may differ when assessed using academic-specific instruments. In contrast, this relationship was attenuated among students with more external control orientations. This pattern suggests that confidence in one’s abilities is most motivational when accompanied by the belief that effort will lead to desired academic outcomes. This distinction, initially proposed by Bandura (1997), is supported by more recent work showing that efficacy expectations and beliefs about outcome controllability jointly predict students’ motivation and effort regulation (Komarraju & Nadler, 2013).

Our findings contribute to the literature in several ways. First, they advance personality theory by specifying the psychological self-efficacy mechanism through which conscientiousness promotes academic outcomes, supporting process-oriented models (Fleeson & Jayawickreme, 2015) over simple trait–outcome accounts. Second, they extend self-efficacy theory by demonstrating that the motivational impact of efficacy beliefs depends on the attributional context, with internal control beliefs amplifying the effect. Third, they integrate personality psychology and educational psychology by illustrating how stable dispositions and malleable beliefs jointly influence engagement, thereby responding to calls for comprehensive models of academic motivation (Dweck, 2017; Yeager & Dweck, 2020).

The practical implications of this study are noteworthy. The mediation results suggest that interventions to enhance engagement should target not only conscientious behaviors but also self-efficacy. Study skills programs may be insufficient unless they provide structured mastery experiences that build confidence in capabilities. Project-based learning approaches, in which students engage with authentic real-world problems through collaborative inquiry, may catalyze active engagement and support the development of higher-order cognitive skills (Rehman et al., 2025). Such instructional strategies could exemplify how principles of mastery-based efficacy-building might be translated into concrete classroom practice. The moderation results further suggest that efficacy-building interventions are most effective when paired with attributional retraining designed to strengthen control beliefs. Students who doubt that their efforts have an influence on outcomes may not fully benefit from enhanced self-efficacy, as external control orientations can undermine motivation. Accordingly, comprehensive engagement interventions should address both capabilities and control beliefs as interconnected components of a coherent motivational system.

This study has some limitations. The cross-sectional design precludes definitive causal inferences; longitudinal designs are required to confirm the temporal ordering of conscientiousness, self-efficacy, and engagement, and assess how these relationships evolve across developmental stages. All focal variables were measured through self-report from the same respondents using the same response format at a single time point, a configuration that is known to elevate the risk of common method variance and inflate observed relationships among constructs. Although Harman’s single-factor test did not indicate a dominant method factor, this diagnostic has well-documented limitations and should not be interpreted as a definitive test of method bias. The observed strength of the correlations among self-report measures, particularly between self-efficacy and academic engagement, may partially reflect shared-method variance rather than exclusively signify substantive relationships. Future studies should employ procedural and statistical remedies for method bias. These include temporal separation of predictor and criterion measurement, the use of multiple data sources (e.g., peer reports, instructor ratings, academic records, behavioral observations), along with more rigorous statistical diagnostics such as the unmeasured latent-method construct approach or marker-variable techniques. The sample, drawn from a single university, limits generalizability across educational levels, cultural contexts, and age groups. Given cross-cultural variations in personality expression and educational values (Henrich et al., 2010), replication across diverse settings is essential. Additionally, all participants were recruited from a single general-education elective course, “Modern Society and Psychology,” which, while open to students across diverse academic majors, may have introduced selection bias. Students who voluntarily enroll in a psychology-adjacent elective may differ from the broader undergraduate population in terms of academic motivation, conscientiousness, or interest in self-reflection. Furthermore, the power imbalance inherent in the instructor–student relationship prevented the collection of personally identifiable demographic information, including age, gender, academic year, or major, per the IRB. This precluded the examination of these variables as potential covariates and limited the evaluation of sample representativeness. Beyond sample composition, the educational context itself may further constrain the broader applicability of the findings. Because all participants were enrolled in the same course and completed the survey within an instructional setting, shared contextual features—including course content, instructor expectations, and the framing of participation as a learning experience—may have systematically shaped how students interpreted and responded to the survey items. These contextual influences could have heightened the salience of constructs such as self-efficacy and academic engagement in ways specific to this instructional environment, potentially inflating their observed associations. Consequently, the present findings should be interpreted as reflecting patterns of association within a particular educational context rather than universal relationships, and caution is warranted when generalizing the findings to students in different instructional settings, course types, or educational cultures.

Accordingly, the present findings should be interpreted as limited to this specific context and not generalizable to broader undergraduate populations or other educational settings. Future studies should recruit participants from a broader range of courses and disciplines and obtain demographic information where ethically feasible. Moreover, the operationalization of academic engagement warrants further consideration. The Kwon (2008) scale captures affective and behavioral dimensions of engagement; however, several items reflect behaviors that extend beyond the motivational core of academic engagement as typically defined in contemporary educational psychology, such as attendance at public lectures and self-directed information-seeking outside formal coursework. These items are better characterized as indicators of broader academic curiosity or enrichment behavior, suggesting that the scale reflects a relatively expansive conceptualization of engagement. This broader operationalization may have introduced conceptual heterogeneity into the outcome variable, potentially attenuating relationships with motivational predictors such as self-efficacy, which are theoretically more proximal to engagement in narrower motivational terms (Fredricks et al., 2004; Skinner et al., 2009). Furthermore, the scale does not fully capture cognitive dimensions of engagement, such as deep-processing strategies and metacognitive regulation, as distinguished by Fredricks et al. (2004). Future research should employ multidimensional instruments that separately assess affective, behavioral, and cognitive dimensions of engagement, grounded in a clearly specified conceptual framework, to enable more precise theoretical conclusions. Finally, the internal consistency of the internal locus of control measure (Cronbach’s α = 0.62) fell below the conventional threshold of 0.70, and the findings should be considered in light of this. In the context of the present study, where internal locus of control functions as the moderator in the central moderated mediation model, this reliability shortfall is particularly consequential. Measurement unreliability in a moderator variable is known to attenuate observed interaction effects because random error reduces the precision with which the interaction term can be estimated (Aguinis et al., 2005; Busemeyer & Jones, 1983). Accordingly, the moderation effects reported here—including the index of moderated mediation and the conditional indirect effects—may underestimate the true strength of internal locus of control as a boundary condition. Low reliability may also affect interpretation of the specific interaction pattern. Measurement error is not distributed uniformly across the latent construct and may distort the functional form of moderator effects. Accordingly, despite the statistically significant moderated mediation result, the specific magnitudes of the conditional indirect effects at low, moderate, and high levels of internal locus of control should be interpreted with caution. Future research should employ more comprehensive and psychometrically robust instruments, ideally domain-specific academic locus of control scales with established reliability above 0.80 to address these issues.

A further limitation concerns the operationalization of internal locus of control. Our theoretical model invokes both generalized and academic-specific control beliefs as relevant to engagement. However, our measure (Levenson, 1973) assesses generalized internal control rather than academic-specific control. Items refer broadly to control over life events rather than specifically to beliefs about academic outcomes (e.g., “I can pretty much determine what will happen in my life”). This mismatch between domain-specific theoretical framing and domain-general measurement raises a construct-validity concern: the moderating effect observed here may underestimate the role that academic-specific control beliefs would play as generalized beliefs are one step removed from the domain in which the outcome is observed. Future research should employ academic locus of control scales (e.g., Trice, 1985) to test whether the moderating effect is stronger, weaker, or structurally different when control beliefs are measured at the same level of specificity as the outcome.

A further limitation concerns the recruitment context. Participants were recruited from a course taught by the research team and informed that participation would serve as a practical learning experience in psychological research methods and that aggregate findings would be shared as part of the course. Participation was voluntary and responses anonymized, with no negative impact if students opted out of the study. However, this recruitment approach may nonetheless have generated subtle pressure to participate or demand characteristics in the response pattern. Future research should recruit participants through channels independent of the research team that decouple study participation from any course-related experience.

In summary, this study provides evidence of patterns of association consistent with a theoretically grounded model in which conscientiousness is indirectly linked to academic engagement via self-efficacy, with the strength of this association varying by levels of internal locus of control. Given the cross-sectional design, these findings should be understood as associations consistent with, rather than proof of, the proposed causal structure. These findings challenge simplistic direct-effect views and highlight a layered psychological architecture in which personality traits operate through proximal beliefs shaped by attributional orientation. By identifying patterns of association linking conscientiousness to engagement, this study offers theoretical insight and practical guidance for developing interventions targeting multiple interconnected components of academic motivation.

## Figures and Tables

**Table 1:** Descriptive statistics of and correlation between the investigated variables.

Variable	Mean	SD	1	2	3	4
1. Conscientiousness	4.66	1.00	-			
2. Internal Locus of Control	4.94	0.68	0.36 **	-		
3. Self-Efficacy	3.53	0.60	0.53 **	0.44 **	-	
4. Academic Engagement	2.38	0.83	0.22 **	0.21 **	0.42 **	-

Note. N = 1059. Correlations were calculated using Pearson’s correlation analysis. ** *p* < 0.01.

**Table 2:** Results obtained in the testing of the mediation of self-efficacy.

Variable	Model 1			Model 2			Model 3		
	Academic Engagement			Self-Efficacy			Academic Engagement		
	*B*(*t*)	β	[95% CI]	*B*(*t*)	β	[95% CI]	*B*(*t*)	β	[95% CI]
Conscientiousness	0.18 (7.29 ***)	0.22	[0.13, 0.23]	0.32 (20.06 ***)	0.53	[0.28, 0.35]	−0.01 (−0.10)	−0.01	[−0.06, 0.05]
Self-efficacy							0.58 (12.91 ***)	0.42	[0.49, 0.67]
*R* ^2^	0.05			0.28			0.18		
*F*	53.20 ***			402.33 ***			114.10 ***		

Note. *B* represents unstandardized coefficients. β = standardized coefficient. 95% CI = bias-corrected confidence interval. *** *p* < 0.001.

**Table 3:** Results obtained in the testing of the moderated mediation model.

Variables	Model 1		Model 2	
	Self-Efficacy		Academic Engagement	
	*B*(t)	[95% CI]	*B*(t)	[95% CI]
Conscientiousness	0.32 (20.06 ***)	[0.28, 0.35]	−0.01 (−0.27)	[−0.06, 0.05]
Self-efficacy			0.57 (12.04 ***)	[0.48, 0.66]
Self-efficacy × Internal locus of control			0.13 (2.67 **)	[0.04, 0.23]
Internal locus of control			0.03 (0.72)	[−0.05, 0.10]
*R* ^2^	0.28		0.18	
*F*	402.33 ***		59.33 ***	

Note. *B* represents unstandardized coefficients. 95% CI = bias-corrected confidence interval. *** *p* < 0.001, ** *p* < 0.01.

## Data Availability

The raw data supporting the conclusions of this article will be made available by the authors on request. Due to ethical restrictions imposed by the institutional review board, the raw data cannot be made publicly available. However, the data can be shared with researchers who meet the criteria for access to confidential data upon request.

## Abbreviations

CI	Confidence interval
VIF	Variance inflation factor
